# Energy Distribution Optimization in Heterogeneous Networks with Min–Max and Local Constraints as Support of Ambient Intelligence

**DOI:** 10.3390/s25092721

**Published:** 2025-04-25

**Authors:** Alessandro Aloisio, Domenico D. Bloisi, Marco Romano, Cosimo Vinci

**Affiliations:** 1 Department of International Humanities and Social Sciences, University of International Studies of Rome (UNINT), 00147 Rome, Italy; domenico.bloisi@unint.eu (D.D.B.); marco.romano@unint.eu (M.R.); 2 Department of Mathematics and Physics “Ennio De Giorgi”, University of Salento, 73100 Lecce, Italy; cosimo.vinci@unisalento.it

**Keywords:** ambient intelligence, multi-interfaces networks, coverage, parameterized complexity

## Abstract

In recent years, ambient intelligence (AmI) has gained significant attention from both academia and industry. AmI seeks to create environments that automatically adapt to individuals’ needs, improving comfort and efficiency. These systems typically rely on Internet of Things (IoT) frameworks, where sensors and actuators enable seamless interaction between people and their surroundings. To ensure the effective operation of AmI systems, robust wireless networks are essential, capable of integrating a wide range of devices across different environments. However, designing such networks presents challenges due to varying communication protocols, power limitations, and the computational capacities of connected devices. This paper introduces a novel approach that leverages multi-interface networks to design a heterogeneous wireless network supporting AmI systems within the IoT ecosystem. The approach centers on selecting the most appropriate communication protocols, such as Wi-Fi, Bluetooth, or 5G, to connect devices. Since many devices are battery-powered, choosing the right communication interface is critical for optimizing energy efficiency. Our primary objective is to improve network performance while extending its operational lifespan by identifying an optimal set of interfaces that balance power consumption and efficiency. We present a new model within the well-established field of multi-interface networks, designed to reduce battery consumption while maximizing network performance. Additionally, we examine the computational complexity of this model and propose two solution algorithms grounded in fixed-parameter tractability theory for specific network classes.

## 1. Introduction

The significant growth of ambient intelligence (AmI) has had an impact both on practical and theoretical studies. The main goal of this research area is to create smart environments that can make decisions in order to adapt and enhance the experience of the user. As technology continues to evolve, the integration of AmI into everyday life becomes increasingly feasible, leading to more intuitive and responsive systems [1]. These systems aim to understand user needs and preferences, ultimately striving to improve comfort, efficiency, and overall satisfaction in various contexts. The potential applications range from smart homes to healthcare settings, showcasing the versatility and importance of this field in shaping the future of human–computer interaction. Additionally, AmI, as well as the Internet of Things (IoT) and artificial intelligence, have shown significant promise in managing emergencies and disasters, highlighting its relevance in critical scenarios [1,2,3,4,5].

AmI systems depend on robust and efficient IoT networks to operate effectively. By utilizing sensors and actuators, IoT facilitates seamless interaction between algorithms and the physical environment [6]. However, integrating diverse devices within IoT networks introduces challenges related to optimizing their interactions. Modern IoT devices are equipped with various communication interfaces, such as Wi-Fi, Bluetooth, 5G, and infrared, each with distinct energy requirements and performance characteristics. While traditional wireless networks typically rely on a single interface, a new area of theoretical computer science involves exploring the benefits of using multiple interfaces to improve the efficiency and performance of IoT networks.

Facilitating the operation of a network made up of diverse IoT devices presents numerous challenges, many of which remain difficult to solve, even in simplified theoretical models. Researchers have devoted significant effort to tackling the complexity inherent in these issues.

Among this class of theoretical problems, we highlight the subclass known as multi-interface network problems, which has revisited many classic graph problems through the lens of using multiple interfaces, protocols, or languages. These problems are also related to graph coloring, although they exhibit both differences and similarities.

In the context of multi-interface network models, one prominent approach is the *coverage* model [7,8,9,10,11,12,13,14,15,16,17,18]. Its primary goal is to ensure that all desired wireless connections within a network are established while minimizing overall energy consumption. Simply put, given an IoT network of heterogeneous devices, each equipped with multiple communication interfaces, the objective is to activate only a subset of these interfaces to enable all required connections, thus reducing the network’s energy cost related to the active interfaces.

Minimizing energy consumption is crucial because wireless devices are typically battery-powered, and prolonging the lifespan of the IoT network requires reducing energy use while still supporting all requested links.

Within the variants of the coverage model already proposed, some of them introduce profits associated with each active interface and each active connection, aiming to provide an incentive to increase the network’s bandwidth. Thus, the problem becomes finding a subset of interfaces that ensures all designated links are maintained, respects the constraints on energy consumption, and maximizes the total profit.

Building on previous approaches, we propose a new coverage model that incorporates both profits and costs. However, instead of limiting the total energy cost, it imposes a constraint based on the maximum energy used by any individual device. Additionally, it introduces a bound on the maximum number of interfaces a device can activate. To implement this bound, we introduce a new condition, that is, the number of interfaces used within each subnetwork, formed by devices within a certain distance of a given device, must be bounded by a value proportional to the number of devices in that subnetwork.

Our model is designed for a wide range of scenarios requiring wireless networks composed of heterogeneous IoT sensors and devices, supporting of AmI frameworks. A relevant real-world example would be an emergency situation, such as a natural disaster (e.g., an earthquake or fire), where standard communication systems may fail. In such cases, an emergency network can be formed using various devices (e.g., laptops, smartphones) and IoT sensors (e.g., smart cameras, temperature sensors, pollution sensors) to relay critical information from the affected area. This heterogeneous IoT network can then be utilized and become part of AmI systems to manage and facilitate disaster recovery [1,2,3,4,5].

### 1.1. Related Work

The field of optimizing device connectivity, particularly with respect to quality of service, has been extensively researched across various perspectives. One research focus in this area involves analyzing how different interfaces are used for specific tasks. For instance, choosing an optimal interface may depend on the size of the file being transferred. In an experimental study presented in [19], researchers compared file transfers over Wi-Fi and Bluetooth across different devices, finding that Wi-Fi was more efficient for larger files, while Bluetooth performed better for smaller ones.

Further research [20,21] examined the feasibility of using two interfaces simultaneously without interference, particularly in relation to thread scheduling. These studies tested the performance of Wi-Fi and LTE interfaces. Another study in a related domain [22] focused on file downloads using both 4G and Wi-Fi.

The potential of using multiple interfaces for file transfer has been explored in [23], where three interfaces were tested individually and in combination. This study covered a range of devices and file types, and the findings suggest that combining 5G and 5 GHz Wi-Fi significantly enhances file transfer performance.

In another approach, ref. [24] investigated concurrent communication across multiple end-to-end paths, introducing a new transport protocol known as multi-path TCP (MTCP). The results demonstrated that using multiple interfaces concurrently reduced communication time and improved fault tolerance.

In the last two decades, multi-interface networks have attracted substantial theoretical interest. Graph theory and network optimization problems often require unique adaptations for multi-interface settings [25]. This research direction began with [26], which introduced the *coverage* problem. This problem aims to identify the most cost-efficient way to establish all potential connections in an undirected graph, where each vertex represents a device and each edge signifies a possible interface-based connection. Variants of the coverage problem have been widely explored [7,8,9,10,11,12,13,14,15,16,17].

Another related problem is edge coloring [27,28,29,30,31], which, while similar to [32], addresses different network constraints. Likewise, the *connectivity* problem seeks the least costly set of connections to maintain full network connectivity, akin to building a minimum spanning tree, with interface costs associated with vertices instead of edges. Research on connectivity and coverage, such as [33,34], also includes minimizing the maximum cost at a single vertex [14].

The *cheapest path* problem, a variation of the classical shortest path problem adapted to multi-interface networks, was examined in [34]. Other significant problems studied in multi-interface networks include *maximum matching* [32] and *flow* [35,36]. These lines of research hold promise for applications in fields such as tactical military operations [37].

### 1.2. Our Results

This paper introduces a novel model for addressing the coverage problem in multi-interface networks. Building on insights from previous work, the goal is to determine a coverage strategy that maximizes overall network profit while imposing two key constraints, namely, limiting the maximum energy consumption per vertex and restricting the number of interfaces used within a local subgraph.

Specifically, we propose a generalized version of the constraint used in [11,12,38,39], which accounts for a subgraph of the original network, rather than focusing on individual vertices, to limit the number of active interfaces. In the original formulation, each vertex had a fixed upper limit of *q* active interfaces. Our new constraint, given an integer input d≥0, restricts the number of active interfaces at vertex *u* to *q* times the sum of one plus the number of vertices within a maximum distance of, at most, *d* from *u*.

This relaxation generalizes the constraint used in [11,12,38,39], meaning that the number of interfaces is no longer strictly tied to individual devices. Instead, the constraint applies to the average number of interfaces within a subnetwork defined by the distance parameter *d*. Compared to previous models [11,12,38,39], our new model allows users to select an appropriate value for *d*, thereby expanding the set of feasible solutions and relaxing the constraint on the number of usable interfaces per vertex. Notably, when d=0, our constraint reduces to the one specified in [11,12,38,39].

The computational complexity of the model derives from the feasibility of the model presented in [11,12,14].

Since the problems turned out to be hard to solve, we decided to tackle them using the framework of parameterized complexity [40,41]. This approach leads to a more fine-grained understanding of the reasons behind such hardness. Compared to classical computational complexity theory, which is one-dimensional, parameterized complexity introduces a second dimension related to a single parameter or a combination of multiple parameters.

After formally introducing the new model, we present positive results, that is, two optimal algorithms specifically designed for series-parallel networks. The first algorithm demonstrates that the problem is fixed-parameter tractable (FPT) [40,41] with respect to the number of available interfaces and maximum energy. The second algorithm establishes fixed-parameter tractability with respect to the number of available interfaces and an upper bound on the maximum profit.

## 2. Preliminaries

For an integer *k*, let [k] denote the set of integers from 1 to *k*, inclusive. In an undirected graph G=(V,E) modeling a network, the set *V* of vertices corresponds to the devices, while the edges, *E*, define the communication links to be established. We denote the maximum degree of the graph by Δ, and the degree of a vertex *u* by δ(u). In the following, we use *k* as the maximum number of different interfaces in the network and [k] as the set of all possible interface types, each identified by an integer from 1 to *k*. We now introduce three functions that will be used in the definition of the problems. They are used to describe the disposition of the interfaces in the network.

**Definition 1.**  *Consider an undirected graph G=(V,E). An availability function is a mapping $\lambda :V\to 2^{\left[k\right]}$ that assigns to each vertex u∈V a subset of [k], such that for every edge {u,v}∈E, the subsets corresponding to u and v have at least one common element.*

In general terms, an *availability function* identifies which interface from the set [k] is accessible at a vertex in the graph, *G*. It ensures that any two connected vertices share at least one interface. This requirement is crucial for ensuring that a feasible solution to the problem exists.

**Definition 2.**  *Given an undirected graph G=(V,E) and an associated availability function λ, an activating function is a mapping $\lambda_{A}:V\to 2^{\left[k\right]}$ where each vertex u∈V is assigned a subset of [k] such that $\lambda_{A}\left(u\right)$ is always contained within λ(u).*

In simpler terms, an *activation function* $\lambda_{A}$ chooses a specific subset of interfaces from those indicated by the availability function λ to be activated on each device within the network.

**Definition 3.**  *In an undirected graph G=(V,E) with a given availability function λ, a feasible activating function is a mapping $\lambda_{A}:V\to 2^{\left[k\right]}$ that assigns to every vertex u∈V a subset of [k], satisfying the following conditions: $\lambda_{A}\left(u\right)$ must be a subset of λ(u), and for every edge {u,v}∈E, the subsets $\lambda_{A}\left(u\right)$ and $\lambda_{A}\left(v\right)$ must have at least one shared element.*

The final definition states that an activating function is deemed feasible if every pair of adjacent vertices selects and activates at least one shared interface type.

Let d≥1 be an integer. We define $\nu_{d}\left(u\right)$ as the number of vertices within a distance of, at most, *d* from a vertex u∈V. Additionally, we denote by $N_{h}\left(u\right)$ the set of vertices at exactly distance *h* from *u*. In particular, when h=1, then $N_{1}\left(u\right)$ corresponds to the usual neighborhood N(u), and we abbreviate $\nu_{1}\left(u\right)$ simply as ν(u).

We are now ready to define the optimization variant of the coverage problem, denoted as *MMCov(q,b,d)*, where the profits on the vertices and edges are given by functions $p:\left[k\right]\to N_{\geq 0}$ and $p:{\left[k\right]}^{2}\to N_{\geq 0}$, respectively.
*MMCov(q,b,d)*: *d*-min–max coverage in multi-interface networks***Input:***A graph G=(V,E), an availability function $\lambda :V\to 2^{\left[k\right]}$ for *G*, an interface cost function $c:\left[k\right]\to N_{>0}$, two integers q,b≥1, an integer d≥0, two profit functions $p:\left[k\right]\to N_{\geq 0}$ and $p:{\left[k\right]}^{2}\to N_{\geq 0}$.***Coverage:***A feasible activating function $\lambda_{A}:V\to 2^{\left[k\right]}$ with respect to λ such that for all u∈V: $|\lambda_{A}\left(u\right)|+\sum_{h\in [d]}\sum_{v\in N_{h}\left(u\right)}\left|\lambda_{A}\left(v\right)\right|\leq \left(1+\nu_{d}\left(u\right)\right)q$ and with $c\left(\lambda_{A}\right)=\max_{u\in V}\sum_{\alpha \in \lambda_{A}\left(u\right)}c\left(\alpha \right)\leq b$.***Task:***Find a coverage $\lambda_{A}$ that maximizes the total profit $p\left(\lambda_{A}\right)=\sum_{u\in V}\sum_{\alpha \in \lambda_{A}\left(u\right)}p\left(\alpha \right)+\sum_{\{u,v\}\in E}\sum_{\alpha \in (\lambda_{A}\left(u\right)\cap \lambda_{A}\left(v\right))}p\left(\alpha ,\alpha \right)$.

**Figure 1:**
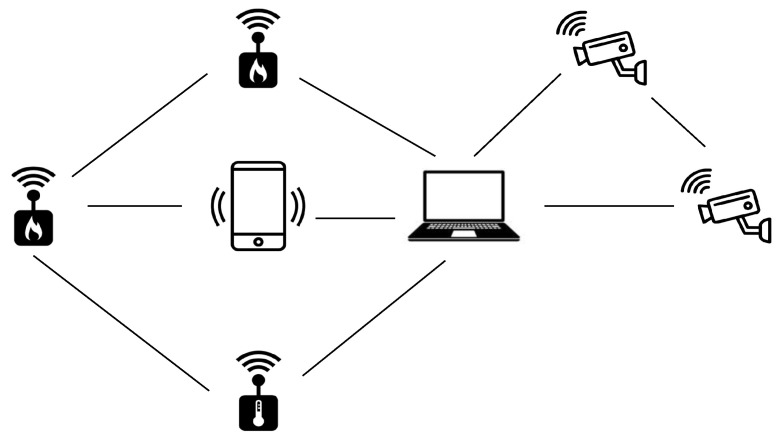
An example of a diverse IoT network integrating devices and smart sensors intended for implementation.

**Figure 2:**
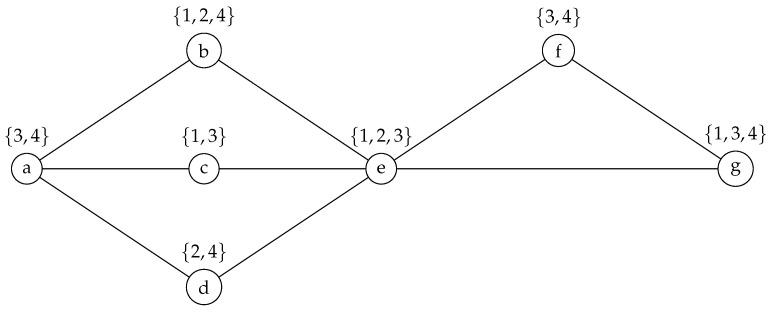
A graph G=(V,E) representing the sample network of Figure 1. Each type of interface is identified by a number between 1 and 4. Specifically, 1 represents Wi-Fi, 2—Bluetooth, 3—Infrared, and 4—5G.

The sample network shown in Figure 1 corresponds to the graph G=(V,E) depicted in Figure 2. For this analysis, we assume k≥2, as the case when k=1 is straightforward, leading to only one possible solution: activating the single available interface across the network.

Previous research has investigated the coverage problem, with particular attention to its computational complexity, in the special case where all costs are uniform and equal to one. This scenario is referred to as the *unitary cost case,* as discussed in [11,12,38,42].

In [12], the authors analyzed the coverage problem without considering profits, focusing instead on minimizing energy consumption, a problem denoted as *CMI(q,∞)*. They proved that this problem is NP-hard. More recent work [38,42] extended this result to models incorporating profits, called *CMI(q,b)*, by demonstrating NP-hardness via a polynomial-time reduction from the classical knapsack problem.

**Theorem 1**  ([38,42]). *CMI(q,b) is NP-hard, even when the input instance admits a feasible solution and q=2.*

We now present a definition of *MMCov(q,b,d)* as a decision problem.
*decMMCov(q,b,d)*: *d*-min–max coverage in multi-interface networks (decision version)***Input:***A graph G=(V,E), an availability function $\lambda :V\to 2^{\left[k\right]}$ for *G*, an interface cost function $c:\left[k\right]\to N_{\geq 0}$, two integers q,b≥1, two integers d,l≥0, two profit functions $p:\left[k\right]\to N_{\geq 0}$ and $p:{\left[k\right]}^{2}\to N_{\geq 0}$.***Coverage:***A feasible activating function $\lambda_{A}:V\to 2^{\left[k\right]}$ with respect to λ such that for all u∈V: $|\lambda_{A}\left(u\right)|+\sum_{h\in [d]}\sum_{v\in N_{h}\left(u\right)}\left|\lambda_{A}\left(v\right)\right|\leq \left(1+\nu_{d}\left(u\right)\right)q$ and with $c\left(\lambda_{A}\right)=\max_{u\in V}\sum_{\alpha \in \lambda_{A}\left(u\right)}c\left(\alpha \right)\leq b$.***Question:***Is there a coverage with profit $p\left(\lambda_{A}\right)=\sum_{u\in V}\sum_{\alpha \in \lambda_{A}\left(u\right)}p\left(\alpha \right)+\sum_{\{u,v\}\in E}\sum_{\alpha \in (\lambda_{A}\left(u\right)\cap \lambda_{A}\left(v\right))}p\left(\alpha ,\alpha \right)$ greater or equal to *l*?

Regarding the computational complexity of the decision variant within our newly proposed coverage model, we can leverage the following result to conclude that *decMMCov(q,b,d)* belongs to the class of NP-complete problems.

**Theorem 2**  ([12]). *Finding a feasible solution for CMI(2,∞) is NP-complete for graphs with Δ≥4, even for the unit cost case and bipartite graphs.*

In fact, for d=0, q=2, a large *b*, and zero profits, finding a feasible solution to our problem is equivalent to finding a feasible solution for *CMI(2,∞)*. Furthermore, *decMMCov(q,b,d)* is in NP, since we can check in polynomial time (with respect to the size of the instance) whether a given activation function respects all the constraints.

The following theorem presents another negative result, suggesting that our model is challenging to solve.

**Theorem 3**  ([14]). *MMCov is NP-hard even when restricted to the bounded unit cost case, for any fixed Δ≥5 and k≥16.*

The model *MMCov*, introduced in [14], aims to find a coverage that minimizes the maximum cost across all devices. This model is closely related to ours when profits are set to zero and constraints on the number of interfaces used locally are removed.

To conclude this section, we want to point out why our constraint concerning the maximum number of interfaces that can be used on each vertex is useful. We recall that when d=0, this constraint turns out to be the one used in [11,12,38,39], and for d≥1, it serves as a relaxation.

It is easy to see how this relaxed constraint can help in finding better solutions compared to those obtained using the classic constraint (d=0), which imposes the same upper bound on the number of interfaces for each device.

As an example, consider a graph G=(V,E) composed of a few high-degree vertices in $V_{1}$, while the remaining devices in $V_{2}$ have low degrees. Let *u* be a vertex in $V_{1}$ with a high degree δ(u). Suppose that, in order to obtain a feasible solution, such a vertex must activate a number of interfaces, $q_{u}$, close to half of δ(u). If we apply the classic constraint (as used in [11,12,38,39]), which enforces the same upper bound, *q*, for all vertices, we must choose *q* to be at least $q_{u}$ in order to obtain a non-empty set of feasible solutions. As a result, even the low-degree vertices in $V_{2}$ would be allowed to activate a large number ($q_{u}$) of interfaces. This leads to a loss of precise control over the total number of interfaces used throughout the network.

In contrast, our new model allows users to impose constraints that are likely to better reflect the structure of the subgraph. For instance, in the graph under consideration, if d=1, we can choose a smaller value for *q*, since for each node $u\in V_{1}$, the upper bound on the number of interfaces used in the star centered at *u* depends on its high degree, δ(u). Thus, we can select *q* such that $\left(q_{u}+\delta \left(u\right)\right)\leq \left(1+\delta \left(u\right)\right)q$. Conversely, for each node $u\in V_{2}$, the upper bound is naturally smaller, as these nodes have low degrees.

### Series-Parallel Graphs

We begin with a brief overview of the definition of series-parallel (SP) graphs.

**Definition 4**  (Series-Parallel Graph). *An SP-graph can consist of a single edge {u,v} with designated terminals i (source) and j (sink). Additionally, if $\hat{G}$ and $\tilde{G}$ are SP-graphs, a new SP-graph can be formed through the following operations:*

- *Series composition: The terminal vertex (sink) of $\hat{G}$ is merged with the starting vertex (source) of $\tilde{G}$.*
- *Parallel composition: The starting vertices (sources) of $\hat{G}$ and $\tilde{G}$ are merged, as are their terminal vertices (sinks).*

SP-graphs without multiple edges are typically called simple SP-graphs. However, since we are not concerned with multiple edges in this context, we will omit the term “simple” for simplicity.

As defined in Definition 4, an SP-graph can be represented using a rooted binary tree structure. In this representation, each leaf node corresponds to a single edge of the SP-graph, while each internal node is labeled either as ‘S’ (indicating series composition) or ‘P’ (indicating parallel composition). This tree-based framework enables the reconstruction of the SP-graph by systematically applying the corresponding series and parallel compositions. Specifically, for an internal node *i*, the subgraph G(i), induced by the edges within the subtree rooted at *i*, is formed by composing two SP-graphs, G(j) and G(h), where *j* and *h* are the children of *i* in the tree.

In [43], the authors demonstrated that the number of edges *m* in an SP-graph can be bounded by the number of vertices *n* in *G*, such that n−1≤m≤2n−3. Additionally, the authors of [44] proposed a linear-time O(n) algorithm to construct the binary tree representation of a graph, *G*, if and only if *G* is an SP-graph.

## 3. Solving *MMCov(q,b,1)* on Series-Parallel Graphs: First Method

Let T be the binary tree representation of an SP-graph G=(V,E). We denote the subtree rooted at node *i* as T(i), and the subgraph of *G* induced by the edges corresponding to the leaves of T(i) as G(i). For a vertex *u*, let $N^{i}\left(u\right)$ denote its neighbors in G(i), and $\nu^{i}\left(u\right)$ denote $|N^{i}\left(u\right)|$. We will now focus on a constrained version of the original problem, which we will leverage to develop a dynamic programming algorithm.
Constrained *MMCov(q,b,1,i,S,T,x,y,$\hat{b}$)*: 1-min–max coverage in multi-interface networks***Input:***A subgraph G(i) induced by the subtree T(i) related to an instance of *decMMCov(q,b,1)* with G=(V,E) has an underlying SP-graph. A subset, *S*, of, at most, $\min\{|\lambda \left(u_{s}\right)|,\left(1+\nu \left(u_{s}\right)\right)q-\nu \left(u_{s}\right)\}$ interfaces of the ones available in terminal $u_{s}$. A subset, *T*, of, at most, $\min\{|\lambda \left(u_{t}\right)|,\left(1+\nu \left(u_{t}\right)\right)q-\nu \left(u_{t}\right)\}$ interfaces of the ones available in terminal $u_{t}$.***Coverage:***A feasible activating function $\lambda_{A}:V\left(i\right)\to 2^{\left[k\right]}$ satisfies the following conditions: for all u∈V(i), $|\lambda_{A}\left(u\right)|+\sum_{v\in N^{i}\left(u\right)}\left|\lambda_{A}\left(v\right)\right|\leq \left(1+\nu \left(u\right)\right)q$, $c\left(\lambda_{A}\right)=\max_{u\in V(i)}\sum_{\alpha \in \lambda_{A}\left(u\right)}c\left(\alpha \right)=\hat{b}$, and the interfaces active on $u_{s}$ and $u_{t}$ are those in *S* and *T*, respectively. Furthermore, the number of interfaces active on $u_{s}$ and $N^{i}\left(u_{s}\right)$ is *x*, while the number of interfaces active on $u_{t}$ and $N^{i}\left(u_{t}\right)$ is *y*.***Goal:***Maximizes the total profit $p\left(\lambda_{A}\right)=\sum_{u\in V(i)}\sum_{\alpha \in \lambda_{A}\left(u\right)}p\left(\alpha \right)+\sum_{\left\{u,v\}\in E(i\right)}\sum_{\alpha \in (\lambda_{A}\left(u\right)\cap \lambda_{A}\left(v\right))}p\left(\alpha ,\alpha \right)$.

At the core of our algorithm, we compute $f(i,S,T,x,y,\hat{b})$, the optimal value of *MMCov(q,b,1,i,S,T,x,y,$\hat{b}$)*, for each node *i* in T. This computation is performed for every possible pair of sets *S* and *T*, where each set contains a number of interfaces bounded above by min{|λ(u)|,(1+ν(u))q−ν(u)}, with $u=u_{s}$ or $u=u_{t}$. Additionally, we consider values *x* and *y* within the ranges $r^{i}\left(u_{s}\right)=\left\{\nu^{i}\left(u_{s}\right)+1,\ldots ,\left(\nu \left(u_{s}\right)+1\right)q-\left(\nu \left(u_{s}\right)-\nu^{i}\left(u_{s}\right)\right)\right\}$ and $r^{i}\left(u_{t}\right)=\left\{\nu^{i}\left(u_{t}\right)+1,\ldots ,\left(\nu \left(u_{t}\right)+1\right)q-\left(\nu \left(u_{t}\right)-\nu^{i}\left(u_{t}\right)\right)\right\}$, respectively. These ranges ensure that at least one interface remains active on $u_{s}$ and $N^{i}\left(u_{s}\right)$ in G(i), while leaving at least one interface available for vertices in $N(u_{s})$ that are not in $N^{i}\left(u_{s}\right)$; an analogous condition holds for $u_{t}$. The procedure initiates at the tree’s leaf nodes and systematically moves upward toward the root of *T*. In cases where no solution matches the specified criteria, the value of $f(i,S,T,x,y,\hat{b})$ is assigned as −∞.

Leaf

We can easily solve our constrained problem when *u* is a leaf by checking the following two cases.

- $f\left(i,S,T,x,y,\hat{b}\right)=\sum_{\alpha \in S}p\left(\alpha \right)+\sum_{\alpha \in T}p\left(\alpha \right)+\sum_{\alpha \in S\cap T}p\left(\alpha ,\alpha \right)$ if
  - S∩T≠∅,
  - $\max\left\{\sum_{\alpha \in S}c\left(\alpha \right),\sum_{\alpha \in T}c\left(\alpha \right)\right\}=\hat{b}$,
  - |S|+|T|=x, and
  - |S|+|T|=y;
- $f(i,S,T,x,y,\hat{b})=-\infty$ otherwise.

These constraints stem from the fact that each leaf *i* in G(i) contains only a single edge. As a result, *S* and *T* must share at least one common interface. Furthermore, *x* and *y* represent the number of active interfaces on $u_{s}$ and $u_{t}$, which correspond to |S| and |T|, respectively. Additionally, the cost is determined by the maximum of $\sum_{\alpha \in S}c\left(\alpha \right)$ and $\sum_{\alpha \in T}c\left(\alpha \right)$.

Interior node labeled with S.

Let *i* be an internal node of T where G(i) corresponds to a series composition of G(j) and G(h), where *j* and *h* are the two children of *i*. Let also $u_{s}$ and $u_{r}$ be the source and sink of G(j), respectively, and $u_{r}$ and $u_{t}$ be the source and sink of G(h). We can determine $f(i,S,T,x,y,\hat{b})$ using the already computed values, and more precisely, by addressing the following problem:

(1) $$\begin{array}{ccc} \max & & f\left(j,S,Z,x,y_{j},{\hat{b}}_{j}\right)+f\left(h,Z,T,x_{h},y,{\hat{b}}_{h}\right)-\sum_{\alpha \in Z}p\left(\alpha \right)\\ s.t. & & \max\left\{{\hat{b}}_{j},{\hat{b}}_{h}\right\}=\hat{b}\\ & & y_{j}+x_{h}-\left|Z\right|\leq \left(1+\nu \left(u_{r}\right)\right)q\\ & & \forall Z\subseteq \lambda \left(u_{r}\right),\;|Z|\leq \min\{|\lambda \left(u_{r}\right)\left|,\left(1+\nu \left(u_{r}\right)\right)q-\nu \left(u_{r}\right)\right\} \end{array}$$

Since, in a series composition, $u_{r}$ is the sink of G(j) and the source of G(h), the optimal value of $f(i,S,T,x,y,\hat{b})$ is given by the sum of $f(j,S,Z,x_{j},y_{j},{\hat{b}}_{j})$ and $f(h,Z,T,x_{h},y_{h},{\hat{b}}_{h})$ minus the profit related to *Z*, for all possible subsets *Z* of the interfaces in $\lambda (u_{r})$, with cardinality at most $\min\{|\lambda \left(u_{r}\right)|,\left(1+\nu \left(u_{r}\right)\right)q-\nu \left(u_{r}\right)\}$. We need to subtract this profit to avoid double-counting. The first constraint ensures the correct energy cost, while the second constraint guarantees that the sum of active interfaces in the star related to $u_{r}$ does not exceed the maximum allowed at distance one. Note that we need to subtract the quantity |Z| to avoid counting it twice in $y_{j}+x_{h}$. There is no need to check this quantity for the remaining vertices, as they are not affected by the series composition.

Interior node labeled with P.

Let G(i) be the subgraph formed by a parallel composition of G(j) and G(h), where *i* is an interior node of T, labeled P, and *j* and *h* are its two children. We can find $f(i,S,T,x,y,\hat{b})$ by solving the following problem, where $u_{s}$ and $u_{t}$ are the source and sink for both G(j) and G(h).

(2) $$\begin{array}{ccc} \max & & f\left(j,S,T,x_{j},y_{j},{\hat{b}}_{j}\right)+f\left(h,S,T,x_{h},y_{h},{\hat{b}}_{h}\right)-\sum_{\alpha \in S}p\left(\alpha \right)-\sum_{\alpha \in T}p\left(\alpha \right)\\ s.t. & & \max\left\{{\hat{b}}_{j},{\hat{b}}_{h}\right\}=\hat{b}\\ & & x_{j}+x_{h}-\left|S\right|=x\\ & & y_{j}+y_{h}-\left|T\right|=y \end{array}$$

The objective function calculates the sum of the optimal profits for a specific pair of subsets, *S* and *T*, representing the active interfaces on the terminals of the subgraphs G(j) and G(h), respectively. Clearly, we need to avoid double-counting the profits associated with both *S* and *T*. The first constraint ensures that the energy cost equals $\hat{b}$. The second constraint limits the number of interfaces used in the star rooted at $u_{s}$ within subgraph G(i) to *x*. Similarly, the third constraint restricts the number of interfaces in the star rooted at $u_{t}$ within subgraph G(i) to *y*. Note that we subtract |S| and |T| because they are counted twice in $x_{j}+x_{h}$ and $y_{j}+y_{h}$, respectively.

Once the algorithm terminates, *decMMCov(q,b,1)* is a yes-instance if and only if there exists at least one value of $f(\mathrm{root},S,T,x,y,\hat{b})$ equal or greater than *l*, where *S* and *T* are subsets of the interfaces in $\lambda (u_{s})$ and $\lambda (u_{t})$ of G(root), with $\left|S|\leq \min\{|\lambda \left(u_{s}\right)|,\left(1+\nu \left(u_{s}\right)\right)q-\nu \left(u_{s}\right)\right\}$ and $\left|T|\leq \min\{|\lambda \left(u_{t}\right)|,\left(1+\nu \left(u_{t}\right)\right)q-\nu \left(u_{t}\right)\right\}$; and $x\in \{\nu \left(u_{s}\right)+1,\ldots ,\left(\nu \left(u_{s}\right)+1\right)q\}$ and $y\in \{\nu \left(u_{t}\right)+1,\ldots ,\left(\nu \left(u_{t}\right)+1\right)q\}$.

Using this algorithm, the optimal value for *MMCov(q,b,1)* is determined as the minimum of $f(\mathrm{root},S,T,x,y,\hat{b})$, evaluated over all possible subsets *S* and *T*, and all possible values of *x* and *y*, subject to the cardinalities and ranges described above.

The pseudocode for the first algorithm is provided in Algorithm 1, where we omit the cardinalities of *S* and *T* for brevity. Here, h(T) represents the height of the tree T and V(T) denotes its set of nodes.
**Algorithm 1:** determining the maximum profits $f(\mathrm{root},S,T,x,y,\hat{b})$.
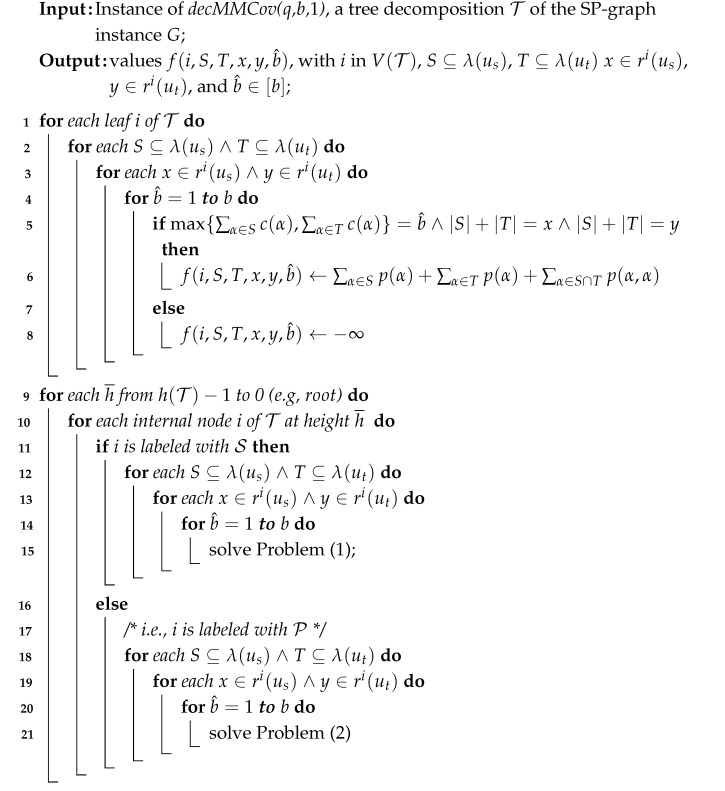

Computational complexity.

For each series composition, the number of instances of Problem (1) that we need to solve depends on the sets *S*, *T*, *x*, *y*, and $\hat{b}$. Once these sets and values are chosen, we must then select *Z*, $y_{j}$, $x_{h}$, ${\hat{b}}_{j}$, and ${\hat{b}}_{h}$. Notably, once any two of $\hat{b}$, ${\hat{b}}_{j}$, and ${\hat{b}}_{h}$ are fixed, the third is determined. Thus, we can assume without loss of generality that we only need to select $\hat{b}$ and ${\hat{b}}_{j}$.

This implies that the number of ways to make these selections is upper-bounded by ∑t∈[(1+Δ)q−Δ]kt3·(Δ+1)4·q4·b2 if (1+Δ)q−Δ≤k, or by $2^{3k}\cdot {\left(\Delta +1\right)}^{4}\cdot q^{4}\cdot b^{2}$, otherwise. This is because each subset, *S*, *T*, and *Z*, is drawn from [k], with cardinality at most min{k,(1+Δ)q−Δ}; each value, *x*, *y*, $y_{j}$, and $x_{h}$ is chosen from [(Δ+1)q], and $\hat{b}$ and ${\hat{b}}_{j}$ are selected from [b].

Once *S*, *T*, *Z*, *x*, *y*, $y_{j}$, $x_{h}$, ${\hat{b}}_{j}$, and $\hat{b}$ are fixed, checking the constraints requires only a constant amount of time. It is easy to verify that the time complexity for a leaf is less than that for an interior node labeled S.

Similarly, for each node labeled P (representing parallel composition), the number of selections of the variables related to Problem (2) is upper-bounded by ∑t∈[(1+Δ)q−Δ]kt2·(Δ+1)4·q4·b2 if (1+Δ)q−Δ≤k, or by $2^{2k}\cdot {\left(\Delta +1\right)}^{4}\cdot q^{4}\cdot b^{2}$ otherwise. This is because there are only two sets, *S* and *T*; we need to choose four of the variables among *x*, *y*, $x_{j}$, $y_{j}$, $x_{h}$, and $y_{h}$, while once again selecting only two from ${\hat{b}}_{j}$, ${\hat{b}}_{h}$, and $\hat{b}$. Here as well, once *S*, *T*, *x*, *y*, $x_{j}$, $y_{j}$, $x_{h}$, $y_{h}$, ${\hat{b}}_{h}$, and $\hat{b}$ are fixed, verifying the constraints takes a constant amount of time.

We are now prepared to present our first main result, noting that the number of nodes in the tree representation of an SP-graph is O(n).

**Theorem 4.**  *Given an instance of decMMCov(q,b,1) with a series-parallel underlying graph, there exists a deterministic algorithm that solves it with a time complexity equal to the minimum between $O(2^{3k}\cdot \Delta^{4}\cdot q^{4}\cdot b^{2}\cdot n)$ and $O(k^{3(1+\Delta )q-3\Delta }\cdot \Delta^{4}\cdot q^{4}\cdot b^{2}\cdot n)$. Since q≤k, the first can be also seen as $O(2^{3k}\cdot \Delta^{4}\cdot k^{4}\cdot b^{2}\cdot n)$.*

**Corollary 1.**  *Given an instance of decMMCov(q,b,1) with a series-parallel underlying graph, there exists an FPT algorithm with respect to the parameter k+b that solves the problem.*

**Corollary 2.**  *Given an instance of MMCov(q,b,1) with a series-parallel underlying graph, there exists a deterministic algorithm that solves it with a time complexity equal to the minimum between $O(2^{3k}\cdot \Delta^{4}\cdot q^{4}\cdot b^{2}\cdot n)$ and $O(k^{3(1+\Delta )q-3\Delta }\cdot \Delta^{4}\cdot q^{4}\cdot b^{2}\cdot n)$. Since q≤k, the first can be also seen as $O(2^{3k}\cdot \Delta^{4}\cdot k^{4}\cdot b^{2}\cdot n)$.*

## 4. Solving *MMCov(q,b,1)* on Series-Parallel Graphs: Second Method

As in the previous section, let T represent the binary tree form of an SP-graph G=(V,E). We denote by T(i) the subtree rooted at node *i*, and by G(i) the subgraph of *G* induced by the edges corresponding to the leaves of T(i). For a vertex *u*, let $N^{i}\left(u\right)$ denote its neighbors in G(i), and $\nu^{i}\left(u\right)$ denote $|N^{i}\left(u\right)|$. First of all, we need to find an upper bound μ to the maximum total profit. Let $p^{*}$ and $p^{**}$ denote the maximum profit obtained on a vertex and an edge, respectively. Since we can activate at most a number of interfaces equal to *q* times the size of each star of the graph, a trivial upper bound is given by $\mu =\min\{\left(q\left(\Delta +1\right)-\Delta \right)\left(np^{*}+mp^{**}\right),k\left(np^{*}+mp^{**}\right)\}$. We are now ready to describe the process.

We now turn our attention to another constrained version of the original problem, which will serve as the basis for developing a dynamic programming algorithm.
Constrained *MMCov(q,b,1,i,S,T,x,y,$\hat{b}$)*: 1-min–max coverage in multi-interface networks***Input:***A subgraph G(i) induced by the subtree T(i) related to an instance of *decMMCov(q,b,1)* with G=(V,E) has an underlying SP-graph. A subset, *S*, of, at most, $\min\{|\lambda \left(u_{s}\right)|,\left(1+\nu \left(u_{s}\right)\right)q-\nu \left(u_{s}\right)\}$ interfaces of the ones available in terminal $u_{s}$. A subset, *T*, of, at most, $\min\{|\lambda \left(u_{t}\right)|,\left(1+\nu \left(u_{t}\right)\right)q-\nu \left(u_{t}\right)\}$ interfaces of the ones available in terminal $u_{t}$.***Coverage:***A feasible activating function $\lambda_{A}:V\left(i\right)\to 2^{\left[k\right]}$ satisfies the following conditions: for all u∈V(i), $|\lambda_{A}\left(u\right)|+\sum_{v\in N^{i}\left(u\right)}\left|\lambda_{A}\left(v\right)\right|\leq \left(1+\nu \left(u\right)\right)q$, $p\left(\lambda_{A}\right)=\sum_{u\in V(i)}\sum_{\alpha \in \lambda_{A}\left(u\right)}p\left(\alpha \right)+\sum_{\left\{u,v\}\in E(i\right)}\sum_{\alpha \in (\lambda_{A}\left(u\right)\cap \lambda_{A}\left(v\right))}p\left(\alpha ,\alpha \right)=\hat{\mu }$, and the interfaces active on $u_{s}$ and $u_{t}$ are those in *S* and *T*, respectively. Furthermore, the number of interfaces active on $u_{s}$ and $N^{i}\left(u_{s}\right)$ is *x*, while the number of interfaces active on $u_{t}$ and $N^{i}\left(u_{t}\right)$ is *y*.***Goal:***Minimizes the cost $c\left(\lambda_{A}\right)=\max_{u\in V}\sum_{\alpha \in \lambda_{A}\left(u\right)}c\left(\alpha \right)$.

At the heart of our algorithm, we compute $g(i,S,T,x,y,\hat{\mu })$ for each node *i* in *T*, representing the optimal value of *MMCov(q,b,1,i,S,T,x,y,$\hat{\mu }$)*. This computation is carried out for every potential pair of sets, *S* and *T*, where each set contains a number of interfaces upper-bounded by min{|λ(u)|,(1+ν(u))q−ν(u)}, with $u=u_{s}$ or $u=u_{t}$. We also consider values *x* and *y* in the ranges $r^{i}\left(u_{s}\right)=\left\{\nu^{i}\left(u_{s}\right)+1,\ldots ,\left(\nu \left(u_{s}\right)+1\right)q-\left(\nu \left(u_{s}\right)-\nu^{i}\left(u_{s}\right)\right)\right\}$ and $r^{i}\left(u_{t}\right)=\left\{\nu^{i}\left(u_{t}\right)+1,\ldots ,\left(\nu \left(u_{t}\right)+1\right)q-\left(\nu \left(u_{t}\right)-\nu^{i}\left(u_{t}\right)\right)\right\}$, respectively, along with each possible $\hat{\mu }\in \left\{0,\ldots ,\mu \right\}$. The algorithm begins at the leaves of the tree and works its way up to the root of *T*.

If there is no solution to the described form, then we set $g(i,S,T,x,y,\hat{\mu })=+\infty$.

Leaf

We can easily solve our constrained problem when *u* is a leaf by checking the following two cases:

- $g\left(i,S,T,x,y,\hat{\mu }\right)=\max\left\{\sum_{\alpha \in S}c\left(\alpha \right),\sum_{\alpha \in T}c\left(\alpha \right)\right\}$ if
  - S∩T≠∅,
  - $\sum_{\alpha \in S}p\left(\alpha \right)+\sum_{\alpha \in T}p\left(\alpha \right)+\sum_{\alpha \in S\cap T}p\left(\alpha ,\alpha \right)=\hat{\mu }$,
  - |S|+|T|=x, and
  - |S|+|T|=y;
- $g(i,S,T,x,y,\hat{\mu })=+\infty$ otherwise.

These constraints arise from the structure of G(i), where each leaf *i* contains exactly one edge. Consequently, *S* and *T* must share at least one common interface. The values *x* and *y* denote the number of active interfaces on $u_{s}$ and $u_{t}$, corresponding to |S| and |T|, respectively. Moreover, the cost is determined by the greater of $\sum_{\alpha \in S}c\left(\alpha \right)$ and $\sum_{\alpha \in T}c\left(\alpha \right)$, while $\hat{\mu }$ must represent the associated profit.

Interior node labeled with S.

Consider an internal node *i* in T, where G(i) represents the series composition of G(j) and G(h), with *j* and *h* being the two children of *i*. Let $u_{s}$ and $u_{r}$ denote the source and sink of G(j), respectively, and $u_{r}$ and $u_{t}$ denote the source and sink of G(h). The value of $g(i,S,T,x,y,\hat{\mu })$ can then be computed based on the previously derived values by solving the following problem:

(3) $$\begin{array}{ccc} \min & & \max\{g\left(j,S,Z,x,y_{j},{\hat{\mu }}_{j}\right),g\left(h,Z,T,x_{h},y,{\hat{\mu }}_{h}\right)\}\\ s.t. & & {\hat{\mu }}_{j}+{\hat{\mu }}_{h}-\sum_{\alpha \in Z}p\left(\alpha \right)=\hat{\mu }\\ & & y_{j}+x_{h}-\left|Z\right|\leq \left(1+\nu \left(u_{r}\right)\right)q\\ & & \forall Z\subseteq \lambda \left(u_{r}\right),\;|Z|\leq \min\{|\lambda \left(u_{r}\right)\left|,\left(1+\nu \left(u_{r}\right)\right)q-\nu \left(u_{r}\right)\right\} \end{array}$$

In summary, for a valid series composition, the vertex $u_{r}$ acts as the sink in G(j) and the source in G(h). Consequently, the optimal value of $g(i,S,T,x,y,\hat{\mu })$ is determined by taking the min–max of $g(j,S,Z,x_{j},y_{j},{\hat{\mu }}_{j})$ and $g(h,Z,T,x_{h},y_{h},{\hat{\mu }}_{h})$, for every subset *Z* that contains at most $\min\{|\lambda \left(u_{r}\right)|,\left(1+\nu \left(u_{r}\right)\right)q-\nu \left(u_{r}\right)\}$ interfaces in $\lambda (u_{r})$. The first condition ensures the correct calculation of profit, but the profit from *Z* needs to be subtracted since it is counted twice. The second condition ensures that the number of active interfaces in the star corresponding to $u_{r}$ does not exceed the allowed limit at a distance of one. There is no need to check this condition for the other vertices, as their interface count remains unchanged in a series composition.

Interior node labeled with P.

Let G(i) represent the subgraph obtained through the parallel composition of G(j) and G(h), where *i* is an internal node of T labeled as P, and *j* and *h* are its two children. The value $g(i,S,T,x,y,\hat{\mu })$ can be determined by solving the following problem, with $u_{s}$ and $u_{t}$ serving as the source and sink for both G(j) and G(h).

(4) $$\begin{array}{ccc} \min & & \max\{g\left(j,S,T,x_{j},y_{j},{\hat{\mu }}_{j}\right),g\left(h,S,T,x_{h},y_{h},{\hat{\mu }}_{h}\right)\}\\ s.t. & & {\hat{\mu }}_{j}+{\hat{\mu }}_{h}-\sum_{\alpha \in S}p\left(\alpha \right)-\sum_{\alpha \in T}p\left(\alpha \right)=\hat{\mu }\\ & & x_{j}+x_{h}-\left|S\right|=x\\ & & y_{j}+y_{h}-\left|T\right|=y \end{array}$$

The objective function returns the maximum of the optimal costs associated with the children *j* and *h*, computed for a specific pair of subsets *S* and *T*, which represent the active interfaces on the terminals of the subgraphs G(j) and G(h), respectively. It also depends on specific values *x* and *y*, which indicate the number of interfaces used in the star graphs of $u_{s}$ and $u_{t}$, as well as a given profit value $\hat{\mu }$.

The first constraint ensures that the profit equals $\hat{\mu }$. The second constraint limits the number of interfaces used in the star rooted at $u_{s}$ to *x*, while the third constraint restricts the number of interfaces in the star rooted at $u_{t}$ to *y*. Note that we need to subtract |S| and |T| because they are counted twice in $x_{j}+x_{h}$ and $y_{j}+y_{h}$, respectively.

Using this algorithm, the instance of *decMMCov(q,b,1)* is a yes instance if there exists at least one value of $g(root,S,T,x,y,\hat{\mu })$ less than or equal to *l*, for two possible subsets *S* and *T* of the interfaces available at the source $u_{s}$ and the sink $u_{t}$ of the root node T; and for two values *x* and *y* range over $\left\{\left(\nu \left(u_{s}\right)+1\right),\ldots ,\left(\nu \left(u_{s}\right)+1\right)q\right\}$ and $\left\{\left(\nu \left(u_{t}\right)+1\right),\ldots ,\left(\nu \left(u_{t}\right)+1\right)q\right\}$, respectively. Clearly the maximum profit $\hat{\mu }$ related to a value (cost) $g(\mathrm{root},S,Z,x,y,\hat{\mu })$ at the root that is less than or equal to *b* is an optimal profit for *MMCov(q,b,1)*.

The pseudocode for the second algorithm is provided in Algorithm 2, with the cardinalities of *S* and *T* omitted for brevity. Here, h(T) represents the height of the tree T, and V(T) denotes its set of nodes.

Computational complexity

For each node labeled S, solving Problem (3) requires making a number of choices that is at most ∑t∈[(1+Δ)q−Δ]kt3·(Δ+1)4·q4·(μ+1)2, if (1+Δ)q−Δ≤k, and $2^{3k}\cdot {\left(\Delta +1\right)}^{4}\cdot q^{4}\cdot {\left(\mu +1\right)}^{2}$, otherwise. This bound arises because we select subsets *S*, *T*, and *Z* from all possible subsets of [k], with cardinality less or equal to min{|λ(u)|,(1+ν(u))q−ν(u)}, with u∈V; values *x*, $y_{j}$, $x_{h}$, and *y* from [(Δ+1)q]; and two values among $\hat{\mu }$, ${\hat{\mu }}_{j}$, and ${\hat{\mu }}_{h}$ from {0,…,μ}.

Similarly, for each node labeled P in Problem (4), the number of choices is at most ∑t∈[(1+Δ)q−Δ]kt2·(Δ+1)4·q4·(μ+1)2 if (1+Δ)q−Δ≤k, and $2^{2k}\cdot {\left(\Delta +1\right)}^{4}\cdot q^{4}\cdot {\left(\mu +1\right)}^{2}$ otherwise, as there are only two sets involved, namely, *S* and *T*.

Since an SP-graph has, at most, 2n−3 edges, the number of nodes in T is O(n). This leads us to the following conclusions.

**Theorem 5.**  *For an instance of decMMCov(q,b,1) with a series-parallel underlying graph, there exists a deterministic algorithm that solves it with a time complexity equal to the minimum between $O(2^{3k}\cdot \Delta^{4}\cdot q^{4}\cdot \mu^{2}\cdot n)$ and $O(k^{3(1+\Delta )q-3\Delta }\cdot \Delta^{4}\cdot q^{4}\cdot {\left(\mu +1\right)}^{2}\cdot n)$. Since q≤k, the first can be also seen as $O(2^{3k}\cdot \Delta^{4}\cdot k^{4}\cdot \mu^{2}\cdot n)$.*

**Corollary 3.**  *Given an instance of decMMCov(q,b,1) with a series-parallel underlying graph, there exists an FPT algorithm with respect to the parameter k+μ that solves it.*

**Corollary 4.**  *For an instance of MMCov(q,b,1) with a series-parallel underlying graph, there exists a deterministic algorithm that solves it it with a time complexity equal to the minimum between $O(2^{3k}\cdot \Delta^{4}\cdot q^{4}\cdot \mu^{2}\cdot n)$ and $O(k^{3(1+\Delta )q-3\Delta }\cdot \Delta^{4}\cdot q^{4}\cdot {\left(\mu +1\right)}^{2}\cdot n)$. Since q≤k, the first can be also seen as $O(2^{3k}\cdot \Delta^{4}\cdot k^{4}\cdot \mu^{2}\cdot n)$.*

**Algorithm 2:** determining the minimum cost values $g(\mathrm{root},S,T,x,y,\hat{\mu })$.

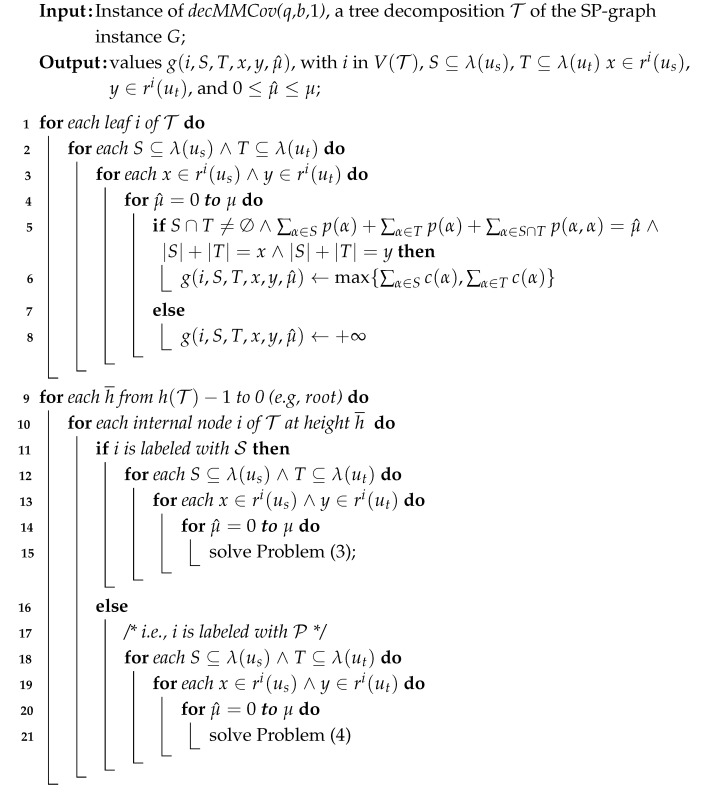



## 5. Conclusions

In this paper, we introduce a novel model within the class of coverage problems for multi-interface networks, designed to support the operation of wireless networks composed of heterogeneous IoT devices—a fundamental component of AmI environments.

Building on established research, we propose a model that considers both energy consumption and incentives for enhancing connection performance. A key innovation is the ability to control the number of interfaces that can be locally activated within specific areas of the wireless network. This approach aims to balance network energy usage and extend battery life while being less restrictive than previous models. Notably, our model allows for flexibility in selecting the size of the subgraph where the number of active interfaces is limited. In fact, by appropriately choosing a parameter, the user can determine the size of this subgraph, whereas in previous models, it was restricted to a single vertex.

We focus particularly on scenarios where restrictions on the number of active interfaces apply to the star rooted at each vertex in the network. Due to the complexity of this model, we specifically analyzed its behavior on series-parallel graphs. We provide two optimal algorithms—one considers the energy budget, and the other considers an upper bound on total profit. The complexity of these algorithms is examined through the lens of parameterized complexity, based on two key parameters, namely, the number of available interfaces combined with the maximum energy budget, and the number of available interfaces combined with the profit upper bound.

Building on this model, we believe further research could explore other graph classes and parameters. Additionally, a promising direction involves a decentralized approach, where each device acts as an agent aiming to maximize its own utility. This scenario introduces a game-theoretic dimension, which could be analyzed using standard metrics like the price of anarchy and the price of stability, along with examining the existence of Nash equilibria. In particular, when budget constraints are removed, the model aligns with polymatrix games and related variants [45,46,47,48,49].

## Data Availability

The original contributions presented in this study are included in the article. Further inquiries can be directed to the corresponding author.
